# Microbiological aspects of osteomyelitis in veterinary medicine: drawing parallels to the infection in human medicine

**DOI:** 10.1080/01652176.2021.2022244

**Published:** 2022-01-02

**Authors:** Margarita González-Martín, Vanessa Silva, Patricia Poeta, Juan Alberto Corbera, María Teresa Tejedor-Junco

**Affiliations:** a Research Institute of Biomedical and Health Sciences, University of Las Palmas de Gran Canaria, Las Palmas de Gran Canaria, Canary Islands, Spain; b Department of Clinical Sciences, University of Las Palmas de Gran Canaria, Las Palmas de Gran Canaria, Canary Islands, Spain; c Microbiology and Antibiotic Resistance Team (MicroART), Department of Veterinary Sciences, University of Trás-os-Montes and Alto Douro (UTAD), Vila Real, Portugal; d Associated Laboratory for Green Chemistry (LAQV-REQUIMTE), University NOVA of Lisboa, Caparica, Portugal; e Veterinary and Animal Research Centre, Associate Laboratory for Animal and Veterinary Science (AL4AnimalS), University of Trás-os-Montes and Alto Douro (UTAD), Vila Real, Portugal; f Department of Animal Pathology, Animal Production and Food Science and Technology, University of Las Palmas de Gran Canaria, Las Palmas de Gran Canaria, Canary Islands, Spain

**Keywords:** Osteomyelitis, *Staphylococcus aureus*, antibiotics, biofilm

## Abstract

Osteomyelitis is a challenging infectious disease affecting humans and animals. It is difficult to diagnose because, in many cases, symptoms are non-specific and, for example in implant-related cases, can appear long time after surgery. In addition to this, it is also difficult to treat due to the need to find the appropriate antibiotic regime and delivery system to reach the site of infection and to avoid development of bacterial resistance. The central purpose of this review is to compare the microbiological aspects of osteomyelitis in human and veterinary medicine, with the aim of improving the microbiological diagnosis and treatment of this infection in animals. Furthermore, the study of osteomyelitis in animals may help to improve the development of animal models for testing new treatments in humans. Host factors and underlying conditions have been studied mainly in humans, although aspects as immunodeficiency have been described in some veterinary cases. Even when *Staphylococcus aureus* is still considered the most prevalent causing microorganism, this prevalence should be reviewed using molecular diagnostic techniques, and this could affect treatment options. New approaches to treatment include local delivery of antibiotics using different biomaterials, antimicrobial photodynamic therapy, and new antimicrobial compounds. We would like to remark the need of large, high-quality clinical trials and of the development of guides for the diagnosis and treatment of osteomyelitis in different animal species.

## 1. Introduction

Osteomyelitis is an infectious disease affecting bone and bone marrow. Kavanagh et al. (2018) summarize the main classification systems used for the diagnosis of osteomyelitis. We have followed the ‘Waldvogel classification’ for this review.

Acute or chronic osteomyelitis can be defined according to the duration of the infection, clinical signs, and response to treatment. Clinical signs of acute osteomyelitis are present for less than 2 weeks and usually respond to antimicrobial treatment alone (Lew and Waldvogel 2004; McNally and Nagarajah 2010). Chronic osteomyelitis evolves over a longer time (months to years) and it is often accompanied by bone destruction and sequestrum formation. In addition, vascular channels are compressed, and an avascular zone is created. The resulting ischemia contributes to bone necrosis and make it more difficult for the immune system and antimicrobials to reach the microorganisms causing the infection (Lew and Waldvogel 2004; Conterno and Turchi 2013). Treatment includes prolonged antimicrobial therapy and is often based on different biomaterials used as local drug delivery systems (Bhattacharya et al. 2013; Caplin and García 2019). Surgical measures are often necessary to remove infected bone (McNally and Nagarajah 2010).

Infection usually reaches the bone by one of two main routes: exogenous or hematogenous. Exogenous osteomyelitis may occur due to fractures or replacement surgery (Hofstee et al. 2020). In humans, exogenous osteomyelitis secondary to vascular insufficiency is also frequently described (Lew and Waldvogel 2004). Hematogenous osteomyelitis is mostly found in paediatric human patients (Conterno and Turchi 2013), but is also described in elderly patients (Lew and Waldvogel 2004; McNally and Nagarajah 2010). In veterinary patients, exogenous osteomyelitis mainly results from direct contamination, either after trauma or surgery (Gieling et al. 2019). Other causes of osteomyelitis in animals are road traffic accidents, gunshot injuries and bite wounds (especially in cats) (May 2002). Hematogenous osteomyelitis is also more common in young animals (Rousseau et al. 2013; Gieling et al. 2019).

The incidence of chronic osteomyelitis in humans is increasing probably due to an aging population, increased prevalence of diabetes and obesity, and more frequent joint replacement surgeries (McNally and Nagarajah 2010).

Osteomyelitis in veterinary patients is difficult to diagnose and treat. Reports in domestic animals are increasing, mainly due to advances in veterinary orthopaedics (Gieling et al. 2019). Cases are also increasingly described in wildlife (Goertz et al. 2011; Konjević et al. 2011; Azorit et al. 2012; Walker et al. 2018; Cotts et al. 2019; Kierdorf et al. 2019).

Main differences in treatment strategies between human and veterinary medicine have economic causes. Long courses of antibiotics and other drugs, surgery and new drug delivery systems are expensive so, in food-producing animals they are not frequently used, unless the patient has a remarkable economic value. These treatments tend to be more frequent in companion animals, including horses. The antibiotic treatment of choice also differs between humans and animals. Not all antibiotics used in the treatment of osteomyelitis in humans are approved for use in veterinary practice (e.g. vancomycin and teicoplanin).

The establishment of osteomyelitis depends on pathogen and host factors. Among the microbial characteristics, the ability to form biofilms, to evade immune response and to invade tissues are particularly relevant (Scherr et al. 2014; Brandt et al. 2018; Krauss et al. 2019; Muthukrishnan et al. 2019). Host factors in human medicine include replacement surgery, trauma, the presence of underlying conditions such as diabetes or tuberculosis, and immunodeficiency (Lew and Waldvogel 2004; Conterno and Turchi 2013; Farnsworth et al. 2018; Hofstee et al. 2020). In veterinary cases, underlying conditions are not studied in depth. Concurrent soft tissue lesions and reduced host defences have been described as important factors contributing to the development of osteomyelitis (May 2002). Clinical cases of osteomyelitis have been described in immunocompromised dogs (Hilligas et al. 2014) or cats (Lo et al. 2012).

The pathophysiology of osteomyelitis varies between animal species. The aim of this review is to analyse microbiological aspects of this infection in humans and veterinary cases.

## 2. Exogenous osteomyelitis

### 2.1. Causes in human medicine

In exogenous osteomyelitis, micro-organisms from host skin or environmental sources are usually the causative agents. In fracture treatment, bone infection can occur by direct contamination through penetrating injuries or during the subsequent surgery. It is usually a polymicrobial infection (McNally and Nagarajah 2010).

In humans, differences have been found between the incidence of implant-associated posttraumatic osteomyelitis depending on whether the surgery was emergency or elective. If emergency surgery was performed on open fractures, the incidence exceeds 30% (Trampuz and Zimmerli 2006; Metsemakers et al. 2018). In elective joint replacements, the incidence ranged from 0.3% to 1.6% (Pulido et al. 2008).

In diabetics, osteomyelitis occurs mainly in the bones of the feet, due to spread from diabetic ulcers (Kavanagh et al. 2018).

### 2.2. Causes in veterinary medicine

Fracture repair in animals is, in some cases, economically unfeasible, so little information can be found about osteomyelitis in some species.

**Figure 1.**
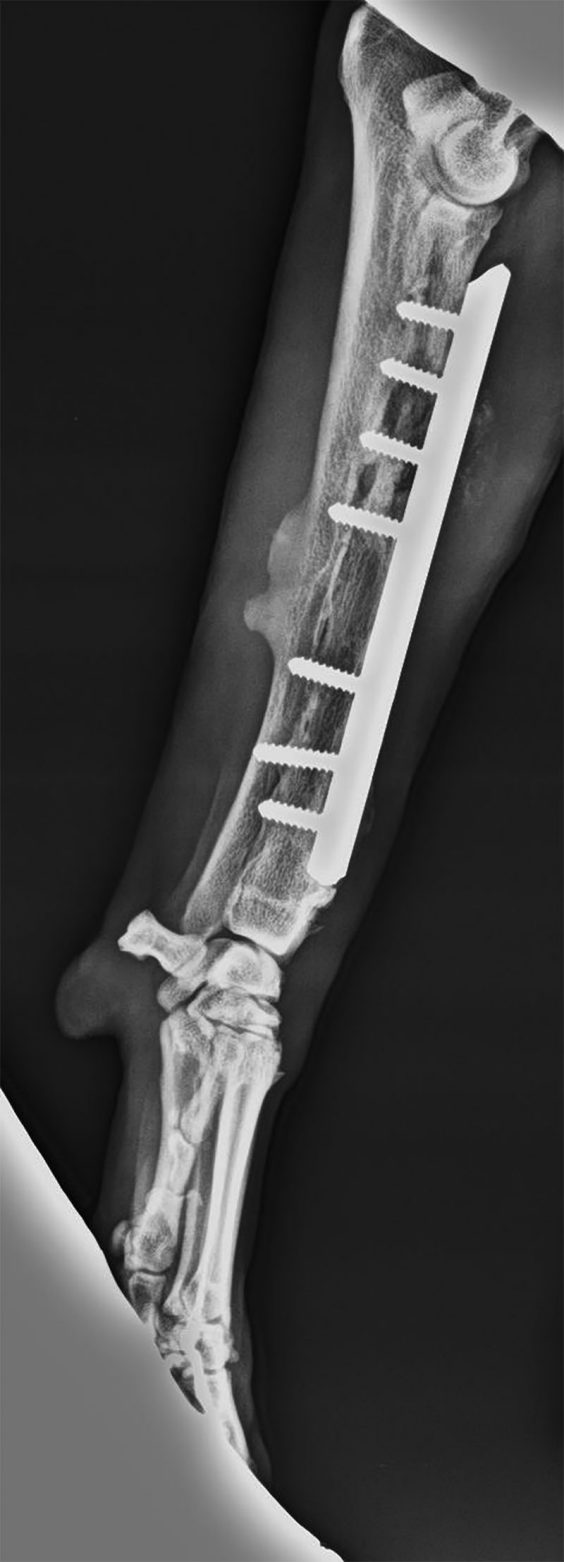
Typical radiological changes from osteomyelitis in a plate implant. Soft tissue swelling, periosteal thickening, lytic lesions, loss of trabecular architecture, new bone apposition and sinus tract formation is observed.

High infection rates, 31% in dogs (Hunt et al. 1980), 28% in horses (Ahern et al. 2010), have been reported in veterinary patients following fracture repair. Also, the incidence of bacterial colonisation of the removed plate implants has been reported (Slunsky 2017). Figure 1 shows the radiological changes caused by osteomyelitis secondary to an implant for fracture repair. Among New World camelids a lower percentage (12%) than in horses has been described by some authors (Knafo et al. 2012), but others have found a similar one (20%) (Semevolos et al. 2008). Fournet et al. (2018) found an infection rate of 7% in feline olecranon fractures.

### 2.3. Maxillomandibular osteomyelitis in human medicine

In humans, bacteria causing maxillomandibular osteomyelitis usually originate from the skin, oral cavity or paranasal sinuses (Krakowiak 2011). The most common are *Staphylococcus aureus*, *S. epidermidis*, *Actinomyces (Trueperella)* and *Prevotella*. It is more frequent in patients with vascular insufficiency or immunosuppression, or with other underlying conditions such as diabetes mellitus or sickle cell anaemia. Malnutrition, high alcohol consumption or intravenous drug use are also important risk factors. Especially remarkable is the high risk of developing maxillomandibular osteomyelitis in patients who have received chemotherapy with bisphosphonates or denosumab (Hofstee et al. 2020), or those who have undergone radiotherapy. The suppurative form is usually more aggressive than the non-suppurative form.

### 2.4. Maxillomandibular osteomyelitis in veterinary medicine

Mandibular osteomyelitis, usually caused by *Actinomycetes*, has been reported in wild and domestic ruminants. It is probably due to bone penetration of the bacteria through primary trauma to the oral cavity, due to sharp, hard forage. The chronic form of the disease causes bone sclerosis, deformity and mandibular swelling (lumpy jaw). As a consequence of pain and difficulties in chewing feed, low body condition appears and even increased mortality has been demonstrated (Konjević et al. 2011; Azorit et al. 2012). Osteomyelitis of the hyoid bones has been described in calves following mandibular injuries (Nuss et al. 2015).

Mandibular osteomyelitis is also frequently described in cats (de Farias et al. 2012; Soto et al. 2014; Bell and Soukup 2015) and dogs (Block and Battig 2017). Bacterial and fungal infections could be involved in both, traumatic and non-traumatic mandibular osteomyelitis. It has been suggested (de Farias et al. 2012) that it may occur secondary to periodontitis or traumatic gum wounds, resembling the process in livestock.

Latney et al. (2016) reported a clinical case of maxillary and premaxillary osteomyelitis in a reticulated python. *Staphylococcus sciuri*, *Enterococcus faecalis* and *Stenotrophomonas maltophilia* were isolated from cultures of the resected maxillary bone.

Clinical cases affecting the sphenoid bone and causing visual impairment in cats and dogs have been described (Busse et al. 2009).

## 3. Hematogenous osteomyelitis

### 3.1. Causes in human medicine

Acute hematogenous osteomyelitis is a common invasive infection in paediatric patients (Conterno and Turchi 2013; McNeil 2020). The pathogenesis is unclear, but it is thought that slow blood flow in the site combined with transient bacteriemia may be the origin (McNeil 2020).

Hematogenous osteomyelitis use to be monomicrobial. *S. aureus* is usually described as the most frequent bacterium causing osteomyelitis, but since 1980s, reports on *Kingella kingae* osteomyelitis have increased markedly and it is now the predominant cause of this infection in young children in Europe (Juchler et al. 2018). Many of the cases were reported as hematogenous osteomyelitis. In a more recent publication (McNeil 2020), *K. kingae* is described as predominant only in the 1-5 years age group, with *S. aureus* being responsible for about 60% of all acute hematogenous osteomyelitis in paediatric patients.

### 3.2. Causes in veterinary medicine

Hematogenous osteomyelitis is not common in veterinary medicine (Carlson 1991). It is mostly described in young animals (Gieling et al. 2019), mainly affecting the metaphyseal area due to its vascular pattern (Carlson 1991; Welch et al. 1997).

In commercial broilers worldwide, Bacterial Chondronecrosis with Osteomyelitis (BCO) represents an emerging cause of lameness (Wideman 2016). Growing broilers on elevated wire flooring induces lameness, frequently associated with BCO of the proximal tibiae and femurs (Al-Rubaye et al. 2017) and, in this study, *Staphylococcus agnetis* was the predominant species isolated and, in some cases, significant bacteriemia was detected. In Australia, Wijesurendra et al. (2017) found that 65% of BCOs were due to *E. coli* with almost all of them being avian pathogenic *E. coli*.

In New World camelids, a study (Rousseau et al. 2013) describes 36 cases of bone sequestration, most of them under of 1 year of age and with no history of trauma. In 10 of the cases, *Fusobacterium* sp. was isolated. The authors propose that haematogenous osteomyelitis could occur in otherwise healthy camelids. The situation resembled that of children and adolescents.

In a 12-months-old heifer, haematogenous osteomyelitis occurred probably due to dissemination after bronchopneumonia (Kofler et al. 2019). A case of vertebral osteomyelitis in a 3.5-month-old heifer appears to be of haematogenous origin as well, but no history of trauma or disease was reported (Shivapour et al. 2019).

### 3.3. Vertebral osteomyelitis in human medicine

Vertebral osteomyelitis (VO) is frequently of hematogenous origin. It usually results in inflammation of the intervertebral disc tissue and adjacent vertebrae. VO accounts for 2-7% of all cases of osteomyelitis and can be classified as tuberculous and nontuberculous depending on its aetiology (Mete et al. 2012). In humans, this condition mainly affects adults over 50 years of age and is difficult to diagnosis as symptoms can be non-specific (Mylona et al. 2009). It usually involves the lower dorsal or lumbar spine. Several microorganisms have been reported as a cause of VO, however *S. aureus* is the most prevalent, being isolated in more than 50% of cases (Go et al. 2012). The second most common organism is *E. coli*, followed by coagulase-negative staphylococci and *Propionibacterium* (Zimmerli 2010; Mete et al. 2012). Although VO is a rare infection in humans, the incidence of this condition is increasing mainly due to growing number of elderly patients and chronic diseases (Gök et al. 2014). However, VO is associated with high morbidity resulting from prolonged antimicrobial therapy and decreased functional status (Doutchi et al. 2015). Skeletal tuberculosis is a form of osteomyelitis due to hematogenous spread of *Mycobacterium tuberculosis*. It can progress slowly over years (Kavanagh et al. 2018).

### 3.4. Vertebral osteomyelitis in veterinary medicine

VO is a well-known condition in food-producing animals and has been reported in several animal species such as cattle, sheep, goats, pigs and horses, as well as in domestic animals (Radostits et al. 2006; Alonso et al. 2019; Shivapour et al. 2019; Giebels et al. 2020; Vieira‐Pinto et al. 2020). However, the traditional name in veterinary medicine for this infection is spondylitis, or discospondylitis if the intervertebral disc is affected (Moore 1992; Thomas 2000).

In food-producing animals suffering from VO, pyaemia may be present at the time of slaughter and the carcasses are declared unfit for human consumption (Vieira‐Pinto et al. 2020). VO is not frequently reported in juvenile cattle. However, some studies have reported VO in calves and foals (Alonso et al. 2019; Lamm et al. 2021). VO in foals often develops secondary to other conditions such as brucellosis or tuberculosis (Alonso et al. 2019). VO in cattle causes weakness, ataxia and recumbency, and has been associated with a wide range of microorganisms such as *Streptococcus epidermidis*, *Fusobacterium spp.*, *Clostridium perfringens, Salmonella* Dublin*, Pseudomonas spp., Escherichia coli* and *Aspergillus fumigatus* (Shivapour et al. 2019).

VO is more frequently detected in broilers and has been reported in several countries worldwide (Wood et al. 2002; De Herdt et al. 2009; Gingerich et al. 2009; Stalker et al. 2010; Kense and Landman 2011; Kolbjørnsen et al. 2011; Makrai et al. 2011; Dinev 2013; Aitchison et al. 2014; Braga et al. 2016; Talebi et al. 2016).

In broilers, VO is an emerging disease causing significant economic losses worldwide. The most common pathogen isolated from VO, as well as from other infections such as spondylitis and arthritis in broilers, is *Enterococcus cecorum* (Makrai et al. 2011; Aitchison et al. 2014). However, recent studies have shown that VO in broilers can also be caused by other agents, such as *E. faecalis*, *E. hirae*, *E. coli* and *S. aureus* and may also involve more than one microorganism (Braga et al. 2016; 2016). This infection occurs most commonly in males and the frequent clinical signs observed are paralysis or paresis that is caused by spinal cord compression resulting from a chronic inflammatory and/or infectious process in the T4 or adjacent vertebrae (De Herdt et al. 2009; Aitchison et al. 2014). Spondylitis may progress to abscess formation, with variable amounts of caseous necrotic material. In some cases, broilers also show an infectious process in the femoral head and pelvic joints (Wood et al. 2002; Martin et al. 2011). In VO outbreaks, broilers at five to eight weeks of age show paralysis of the pelvic limbs. The mortality rate in VO outbreaks ranges from 5 to 15%, mainly due to difficult access to the feeders and drinkers (Borst et al. 2015, 2017).

**Figure 2.**
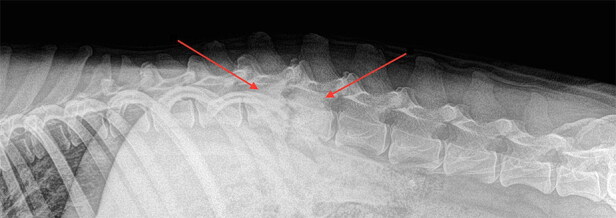
Common radiographic findings associated with discospondylitis. Osteolysis of vertebral end plates and adjacent vertebral bodies with collapse of the intervertebral disk space is observed between L1 and L2.

However, in relation with the small animals, *Staphylococcus* species are the most common pathogens identified in dogs diagnosed with discospondylitis. *Streptococcus* species, *Escherichia coli*, *Aspergillus* species, and *Brucella canis* or *Brucella suis* has been isolated less frequently (Fischer et al. 1997; Buhmann et al. 2019). Spinal pain is the main clinical sign and clinical imaging (radiography and computed tomography) is a frequently used screening method (Figure 2) (Ruoff et al. 2018). In cats, discospondylitis is uncommonly diagnosed (Packer et al. 2005).

## 4. Microbiological aspects

As a general principle, for diagnosis, it is important to consider the whole patient and not just the bone infection. The same principle should apply to the choice of treatment (McNally and Nagarajah 2010).

For laboratory diagnosis of osteomyelitis, especially when related to prosthetic replacement, five or more deep tissue/implant samples should be submitted to the laboratory. In case of suspected fracture-related infection, an antibiotic should be administered after sampling to prevent the inhibition of the growth of microorganisms in cultures. In cases of chronic osteomyelitis, antibiotic treatment should be withdrawn for two weeks prior to sampling (May 2002). False positive (due to commensal micro-organisms) and false negative (due to growth of biofilms or intracellular bacteria) results are frequent (Govaert et al. 2020). Swabs should not be used for sampling due to worse results compared to tissue cultures (Aggarwal et al. 2013). It is necessary to disrupt biofilms (sonication or vortexing), use enrichment media and maintain cultures long enough to allow the detection of slow-growing bacteria. Enrichment broths should be subcultured as soon as turbidity is observed. Incubation should be maintained for at least 5-7 days before they are considered negative (Govaert et al. 2020). Due to the wide variety of possible microorganisms implied and the different detection methods used in the laboratories, it is difficult to establish more specific timelines. In addition to this, longer periods of incubation also increase the possibility of false positives due to the growth of commensal bacteria. Maintaining cultures for 14 days should be enough to detect most of slow growing microorganisms, including fungi. Molecular techniques, like real time PCR, could help in guiding the diagnosis.

Fine needle aspiration or bone biopsies have been used for sampling in paediatric osteomyelitis and in vertebral osteomyelitis. Confirmation of *Mycobacterium tuberculosis* osteomyelitis requires acid-alcohol resistance staining of the biopsy or specific culture techniques.

Blood cultures should be taken in case of fever or suspected haematogenous osteomyelitis, but probability of a positive results is higher if cultures of bone exudates, abscesses or aspirates from adjacent joints or soft tissues are used (McNeil 2020). Some fastidious microorganisms, as *Kingella kingae*, are best detected by PCR, but can be isolated by inoculating bone or synovial fluid samples into blood culture bottles. In several studies of acute hematogenous osteomyelitis in children, *K. kingae* has been described as the most frequently isolated microorganism (Juchler et al. 2018).

In acute infection, Gram staining of the specimen can guide the diagnosis allowing rapid treatment, but antimicrobial susceptibility testing should be performed to confirm the susceptibility. Determination of minimum biofilm eradication concentration (MBEC) (Dalecki et al. 2016), in addition to minimum inhibitory concentrations (MICs), could help in deciding the most appropriate antimicrobial therapy.

Molecular detection by PCR is a possibility, but if the microorganism is not cultured, antimicrobial susceptibility testing cannot be performed. In some cases, these techniques allow detection of some antimicrobial resistance genes.

### 4.1. Microbiology of osteomyelitis in humans

In humans, *Staphylococcus aureus* is by far the most common cause of all types of osteomyelitis. Most research on human osteomyelitis has focused on this bacterial genus. Adhesins allow binding to components of the extracellular matrix, mainly fibronectin and collagen. Once colonisation of bone has occurred, *S. aureus* is able to establish chronic infection, surviving within abscesses and spreading through the canicular network, where it can also survive due to difficult access of antimicrobial or the immune system. Invasion and adherence to bone sequestrum (devitalized bone fragments) may also occur (Brandt et al. 2018).

*S. aureus* has several virulence factors that allow hematogenous dissemination and infection of bone. *S. aureus* interactions with receptors of innate immunity alter the bone remodelling activities of osteoblasts and osteoclasts.

In addition, virulence factors induce bone cells death and contribute to the pathogenesis of osteomyelitis (Muthukrishnan et al. 2019). Panton-Valentine leucocidin (PVL) production is associated with a more severe osteomyelitis in children (Brandt et al. 2018). In some animal models, PVL increases bacterial survival in bone and facilitates bacterial spread to nearby muscles and joints. In a murine model of osteomyelitis, toxic shock syndrome toxin 1 (TSST-1) and Protein A activate osteoclast signalling to increase bone resorption. Phenol-soluble modulins (PSMs) are involved in 30% of cortical bone loss with a direct cytolytic effect on osteoblasts.

Biofilm formation on implanted material or necrotic bone is the most important causes of *S. aureus* persistence in osteomyelitis. Mutation of the staphylococcal accessory regulator (*sarA*) causes a significant decrease in biofilm formation and has been proposed as a viable target for biofilm-associated infections such as osteomyelitis (Loughran et al. 2016).

*S. aureus* can also invade and survive inside the cells of the immune system. It also has the ability to infect and replicate within host cells (Loughran et al. 2016; Krauss et al. 2019; Muthukrishnan et al. 2019). This allows bacteria to survive against the immune system and antimicrobial treatment.

### 4.2. Microbiology of osteomyelitis in animals

The study of osteomyelitis in animals may help to improve the development of animal models for testing new treatments in humans. Not all animal models are suitable for testing the effect of *S. aureus* toxins. For example, PVL activity cannot be investigated using murine models because its activity is restricted to human and rabbit C5a receptors. Other staphylococcal toxins also have species-specific interactions with receptors (Brandt et al. 2018). White rabbits are frequently used as models, as are pigs and other animals (Pearce et al. 2007; Schafrum Macedo et al. 2019). Rabbits are considered a reliable and reproducible model of orthopaedic infections. The size of rabbits allows testing the implantation of orthopaedic devices to simulate implant-associated infections in humans (Bottagisio et al. 2019). Rabbits’ immune system is more similar to human’s immune system than rodents’ one. Their susceptibility to infections is similar to that of humans and they are highly susceptible to PVL (Bottagisio et al. 2019). Diabetic mouse and rat strains have been developed to study the impact of this condition in osteomyelitis development (Lovati et al. 2013; Brown et al. 2014). Small animal models are useful because of lower costs, easier handling and the possibility to evaluate a large number of cases. However, efficacy and safety must subsequently be demonstrated in a large animal model (pigs, sheep) before clinical trials in humans (Roux et al. 2021). An updated review of animal models can be found in Wong et al. (2020) and in Roux et al. (2021).

In animals, beta-lactamase-producing *Staphylococcus* species accounts for half of the cases of osteomyelitis. *Streptococcus*, *E. coli* and other *Enterobacterales*, *Pseudomonas aeruginosa*, *Pasteurella*, *Nocardia* and Anaerobes (i.e. *Bacteroides*, *Fusobacterium*, *Actinomyces*) are also frequently described (May 2002; Kaya et al. 2011; Siqueira et al. 2014; Soto et al. 2014; Twitchell et al. 2014; Salas et al. 2015). Among fungi, cases due to *Cryptococcus* (Cazzini et al. 2013; Block and Battig 2017), *Candida* (Doyle et al. 2013), *Blastomyces* (Mendez-Angulo et al. 2011)*, Aspergillus* (Hunter and Nation 2011; Brocal et al. 2018) or *Penicillium* (Langlois et al. 2014) have been described in different animal species.

Even the tick-borne protozoan *Hepatozoon canis* has been found causing osteomyelitis in a dog (Shimokawa Miyama et al. 2011). Silveira et al. (2020) describes a case of a dog with recurrent Urinary Tract Infection (UTI) due to osteomyelitis of the penile bone and proposes that this condition should be included in differential diagnosis of partial and complete urethral obstruction in dogs with recurrent UTI.

As explained above, *S. aureus* is the most commonly isolated bacterium in human osteomyelitis (Kavanagh et al. 2018; Hofstee et al. 2020). *Enterococcus*, *Enterobacterales*, *Streptococcus* and anaerobic bacteria are also frequently detected in acute osteomyelitis (McNally and Nagarajah 2010; McNeil 2020). In polymicrobial infections after injury or surgery, *S. aureus* is de predominant bacteria, but together with coagulase-negative staphylococci, *Propionibacterium acnes* and Gram-negative bacilli. In contaminated open fractures, *Clostridium* and *Nocardia* may contribute to infection (Pulido et al. 2008; McNally and Nagarajah 2010). *Pseudomonas aeruginosa* is also a frequent finding and, due to multi-drug resistance of this species, a major cause of concern (McNally and Nagarajah 2010).

In neonates and infants, hematogenous osteomyelitis is as often caused by Streptococci as by Staphylococci species (Lew and Waldvogel 2004), so appropriate culture media must be selected.

There are many special situations, depending on the host and geographical area. Patients with sickle cell anaemia, immunocompromised patients, drug addicts and, above all, patient with tuberculosis have specific characteristics that must be taken into account when microbiological cultures of osteomyelitis samples are processed in the laboratory.

## 5. Treatments

Different aspects that complicate the management of osteomyelitis include: the fact that the clinicians cannot diagnose the infection until it has reached a chronic stage where the local vasculature is compromised; the formation of biofilms and the emergence of phenotypic variants within them, which limits the efficacy of antibiotics and host defences, and the ability of different bacteria to invade and replicate within host cells. (McNally and Nagarajah 2010; Loughran et al. 2016). These characteristics are particularly relevant in *S. aureus* but have also been studied in other genera as *Pseudomonas*.

The formation of biofilms on the implant surface is the main difficulty in eradicating the infection. Bacteria growing in biofilms are up to 1000 times more resistant to antibiotics than in planktonic state (Depypere et al. 2020). Antimicrobial Photodynamic Therapy (aPDT) has recently been proposed to combat clinically relevant biofilms, including prosthetic joint infections (Hu et al. 2018). This therapy has been used in snakes to treat stomatitis, thereby decreasing the risk of developing osteomyelitis or septicaemia (Grego et al. 2017).

Acute uncomplicated cases of osteomyelitis can be treated with antibiotic therapy alone, usually for 4-6 weeks. The success rate is approximately 80%. In chronic and implant-associated osteomyelitis, antibiotic therapy alone does not yield satisfactory success rates, and surgical treatment (debridement) is often necessary as well (Hofstee et al. 2020). Prompt debridement reduces the bacterial load at the site of infection, increasing the efficacy of antibiotics and reducing the risk of development of antimicrobial resistance. It will also reduce the inflammatory response and the risk of chronification of infection.

Systemic administration of antibiotic in high doses and for prolonged periods can lead to toxic effects. Local delivery of antibiotics using different biomaterials reduces the need for systemic therapy and contributes to prevent relapse (Kavanagh et al. 2018; Caplin and García 2019). Commonly reported delivery vehicles used as local drug carriers for antibiotics are hydrogels, cements, micro- and nanoparticles, coating/films, scaffolding, and sponges. Particularly the new generation ones have proven to be more appropriate to prevent antibiotic resistance events. These new devices permit an initial burst of the therapeutic followed by a slow, low dose elution of residual therapeutic (Cobb et al. 2020). In dogs, gentamicin-impregnated sponges have been used as adjuvant therapy of osteomyelitis (Wainberg et al. 2015). Also, amylose starch implants have been tested as a biodegradable antimicrobial delivery system in dogs for the prevention and treatment of osteomyelitis (Huneault et al. 2004). A macaque with marked osteomyelitis due to a bite wound was successfully treated with polymethylmethacrylate beads impregnated with vancomycin and tobramycin after failure of oral and systemic antimicrobial therapy (Kelly et al. 2012).

New antibiotics, like dalbavancin, have been tested *in vitro* and *in vivo* against biofilm-forming enterococci and staphylococci, showing promise as an antibiotic for the treatment of osteomyelitis (Silva et al. 2020).

The choice of antimicrobial depends on many factors. In general, Methicillin-Resistant *Staphylococcus aureus* infection is treated with intravenous (IV) vancomycin or teicoplanin. Ceftriaxone may be used for other staphylococci or for streptococci infection. Anaerobes may require clindamycin. Treatment of *Pseudomonas* is difficult, but quinolones or aminoglycosides seems the best option. After a few days of intravenous antibiotics, a switch to oral therapy can be made if an improvement in the patient's condition is observed. Antibiotics with high bone penetration are always needed.

Bacteriostatic antibiotics should be avoided. Poor blood supply limits the ability of the immune system to eliminate the infection.

In dogs, high susceptibility rates to amoxicillin-clavulanic acid, ceftiofur, ceftriaxone and ciprofloxacin have been found in osteomyelitis-causing bacteria (Siqueira et al. 2014).

The efficacy of local and systemic antimicrobial agents have been studied in rabbit models of osteomyelitis because they exhibit similar pharmacokinetics to humans (Bottagisio et al. 2019). Helbig et al. (2014) have developed a rabbit model of peri-implant osteomyelitis to study infection and the efficacy of different antimicrobial therapies, especially vancomycin against methicillin-resistant *S. aureus*.

To avoid development of resistance and toxicity, a combination of two drugs should be used whenever possible.

## 6. Conclusions

Osteomyelitis is an infectious disease affecting humans and animals that is difficult to diagnose and treat. Several classifications have been proposed to facilitate clinical decisions, but they are useful mainly for humans.

Cases of chronic osteomyelitis in humans are relatively common, due to the increasing elderly population, the high prevalence of underlying diseases such as diabetes mellitus and the increased frequency of joint replacement surgery.

In veterinary medicine, advances in orthopaedics have represented significant progress for animal welfare, but they have also led to an important increase in veterinary cases of osteomyelitis. Underlying conditions such as diabetes or immunosuppression, described in humans, have not been studied in animal osteomyelitis.

In terms of prevalence by age group, hematogenous osteomyelitis is more frequent in young individuals, both in animals and humans.

Significant advances have been made in the laboratory diagnosis of this disease thanks to molecular techniques. Using techniques such as PCR, more fastidious micro-organisms, such as *K. kingae*, are detected, which raises the need to reconsider the prevalence data of *Staphylococcus aureus*.

There is not a general consensus in either human or veterinary medicine on the duration and method of administration of treatment. Large, high-quality clinical trials are scarce in humans and almost non-existent in veterinary cases.

We therefore believe that guidelines for the treatment of osteomyelitis should be developed that reflect the prevalence of antimicrobial resistant pathogens, the most appropriate drugs and routes of administration, and gold standards for laboratory diagnosis of each type of osteomyelitis, both in humans and in different animal species.
